# Total Exposure Study Analysis consortium: a cross-sectional study of tobacco exposures

**DOI:** 10.1186/s12889-015-2212-5

**Published:** 2015-09-07

**Authors:** Andrew W. Bergen, Ruth Krasnow, Harold S. Javitz, Gary E. Swan, Ming D. Li, James W. Baurley, Xiangning Chen, Lenn Murrelle, Barbara Zedler

**Affiliations:** Center for Health Sciences, SRI International, 333 Ravenswood Avenue, Menlo Park, CA 94025 USA; Stanford Prevention Research Center, Stanford University School of Medicine, Palo Alto, CA 94305 USA; Department of Psychiatry and Neurobehavioral Sciences, University of Virginia, Charlottesville, VA 22911 USA; BioRealm, LLC, Culver City, CA 90230 USA; University of Nevada, Las Vegas, NV 89154 USA; Venebio Group, Richmond, VA 23225 USA

## Abstract

**Background:**

The Total Exposure Study was a stratified, multi-center, cross-sectional study designed to estimate levels of biomarkers of tobacco-specific and non-specific exposure and of potential harm in U.S. adult current cigarette smokers (≥one manufactured cigarette per day over the last year) and tobacco product non-users (no smoking or use of any nicotine containing products over the last 5 years). The study was designed and sponsored by a tobacco company and implemented by contract research organizations in 2002–2003. Multiple analyses of smoking behavior, demographics, and biomarkers were performed. Study data and banked biospecimens were transferred from the sponsor to the Virginia Tobacco and Health Research Repository in 2010, and then to SRI International in 2012, for independent analysis and dissemination.

**Methods:**

We analyzed biomarker distributions overall, and by biospecimen availability, for comparison with existing studies, and to evaluate generalizability to the entire sample. We calculated genome-wide statistical power for *a priori* hypotheses. We performed clinical chemistries, nucleic acid extractions and genotyping, and report correlation and quality control metrics.

**Results:**

Vital signs, clinical chemistries, and laboratory measures of tobacco specific and non-specific toxicants are available from 3585 current cigarette smokers, and 1077 non-users. Peripheral blood mononuclear cells, red blood cells, plasma and 24-h urine biospecimens are available from 3073 participants (2355 smokers and 719 non-users). In multivariate analysis, participants with banked biospecimens were significantly more likely to self-identify as White, to be older, to have increased total nicotine equivalents per cigarette, decreased serum cotinine, and increased forced vital capacity, compared to participants without. Effect sizes were small (Cohen’s d-values ≤ 0.11). Power for *a priori* hypotheses was 57 % in non-Hispanic Black (*N* = 340), and 96 % in non-Hispanic White (*N* = 1840), smokers. All DNA samples had genotype completion rates ≥97.5 %; 68 % of RNA samples yielded RIN scores ≥6.0.

**Conclusions:**

Total Exposure Study clinical and laboratory assessments and biospecimens comprise a unique resource for cigarette smoke health effects research. The Total Exposure Study Analysis Consortium seeks to perform molecular studies in multiple domains and will share data and analytic results in public repositories and the peer-reviewed literature. Data and banked biospecimens are available for independent or collaborative research.

**Electronic supplementary material:**

The online version of this article (doi:10.1186/s12889-015-2212-5) contains supplementary material, which is available to authorized users.

## Background

The Total Exposure Study (TES) was designed by a tobacco company sponsor in the 1990s with the primary objectives of estimating exposure of current U.S. adult cigarette smokers to cigarette smoke constituents and of investigating relationships between FTC tar categories and cigarette smoke exposure. Other objectives included investigating associations of smoking behavior and biomarkers of exposure (BOE), comparing BOE in adult smokers and non-users, and investigating relationships between BOE and biomarkers of potential harm (BOPH) [1]. From 2002 to 2003, internationally-recognized contract research organizations (CROs), under contract to the tobacco company sponsor, collected questionnaire data, clinical data, and biological samples from 3,585 smokers and 1077 non-users at 39 clinical sites in 31 U.S. states and performed clinical chemistry, laboratory and statistical analyses [1–3]. TES participants were recruited using Institutional Review Board-approved advertisements [1, 2], with defined inclusion and exclusion criteria (Additional file 1). The study was approved by an Institutional Review Board at each clinical site and conducted in accordance with Good Clinical and Laboratory Practices and principles of the Declaration of Helsinki. Using blood and urine biospecimens and mass spectrometry-based and clinical chemistry-based analyses, the CROs determined levels of BOE and BOPH in smokers and non-users. Additional blood and urine biosamples were collected from consenting subjects for possible future analyses.

The Virginia Tobacco Health Research Repository (VTHRR) was formed in 2010 as a Virginia non-profit, non-stock corporation by authorization of the Virginia BioTechnology Research Partnership Authority Board, a political subdivision of the Commonwealth of Virginia. The VTHRR received TES data and biospecimens as a contribution from the tobacco company sponsor. The mission of the VTHRR is to make the TES data and banked biospecimens available to scientists, research institutions, regulatory agencies and industry for research to increase the scientific knowledge base of the health effects of cigarette smoking [4].

Under a 2012 Asset Transfer Agreement between the VTHRR and SRI International (SRI), an independent, non-profit research institute incorporated in 1946 in the state of California, TES data and biospecimens were transferred to SRI in 2012. The agreement between SRI and VTHHR provides SRI with complete independence to pursue valid scientific objectives. The principal intended result of any analysis of TES data or biospecimens is the generation of knowledge related to smoking and health that is shared in the scientific peer-reviewed literature and in appropriate databases. SRI will independently maintain, curate, and make both data and biospecimens available to the research community for this purpose.

In order to optimize the validity and utility of the TES data and banked biospecimens to support its full use by the global public health research community, there is a need for thoughtful, objective scientific analysis of the resource. The purpose of this analysis was to review TES data and biospecimens, investigate distributions of self-reported, clinical and laboratory measures of exposure and potential harm (biomarker), and pote`ntial differences in biomarker levels between those participants with banked biospecimens and those without, calculate statistical power for genomic analyses, and perform analyses of plasma and peripheral blood monocyte analytes.

## Methods

We obtained ethical approval from the SRI International Human Subjects Committee to conduct these analyses of TES data and biospecimens.

Each study site selected to use either their individual site-specific IRB or a central IRB contracted by the primary clinical and laboratory CRO responsible for the conduct of the study. TES participants were recruited, provided informed consent and were screened in a two-visit, multicenter process as current cigarette smokers, stratified by their regular cigarette’s Federal Trade Commission (FTC) tar level (≤2.9, 3.0–6.9, 7.0–12.9, and ≥ 13 mg), and as non-users [1, 2]. Inclusion and exclusion criteria are described in Additional file 1. Participants were paid up to 300 U.S. dollars for completion of all study components. Recruitment sites were distributed in 31 States over four regions [Midwest (19.7 %), Northeast (13.0 %), South (37.8 %) and West (29.5 %)] and among urban (68.5 %) and non-urban (31.5 %) areas.

All participants provided vital signs (at both visits), medical history and concomitant medication data (at the first visit), and completed a questionnaire survey regarding smoking history and attitudes and preferences regarding smoking (in current smokers), demographics, lifestyle and environmental exposures (at the second visit). Between the first and second visit, smokers collected cigarette butts over a 24-h period and smoking topography information using a portable instrument which measured the number of puffs, the length of puffs and the length of the inter-puff interval. Both smokers and non-users collected their urine over 24-h. At the second visit, lung function tests were performed and blood was collected for processing, biomarker assays and, under a separate consent for future research, for banking.

Four tubes of whole blood [two 10 ml potassium ethylenediaminetetraacetic acid (KEDTA) and two 8.5 ml acid citrate dextrose solution A (ACDA) tubes] were obtained from each participant at the second visit after a minimum 6 h fast and processed for plasma, red blood cells and monocytes [1]. The TES biospecimen aliquots in SRI’s possession include approximately: a) 6000 peripheral blood mononuclear cell (PBMC) samples; b) 7000 red blood cell samples; c) 5000 24-h urine samples; and d) 3000 plasma samples. TES biospecimens have been stored at −80 °C by the VTHRR and SRI.

We examined TES publications, accessed the University of California San Francisco Legacy Tobacco Documents Library (UCSF LTDL) website [5] TES-related documents to 1) compare with documents we had received from the VTHRR and 2) to learn more about the study design and analysis goals of the TES, and engaged with colleagues regarding the potential value of the TES for tobacco research. We reviewed data collection, sample preservation, and laboratory assay protocols followed by the CROs that conducted the TES. We inspected TES clinical and biospecimen data and labeled biospecimens to confirm that the dataset was deidentified.

We queried the TES clinical data to assess the distribution of participant data among the analysis strata (age, sex, and BMI) among the four smoking categories defined by the smoker’s usual cigarette FTC tar level, and among non-users. We evaluated the distributions of analysis strata among all participants, by banked biospecimen availability, and by biospecimen type. We analyzed additional behavioral, demographic, biomarker and tobacco product variable distributions among participants, and compared distributions between participants with and without banked biospecimens.

We constructed logistic regression models predicting the availability of biospecimens in self-identified non-Hispanic Black and White smokers using individuals with complete data in three increasingly complex models. Model 1 comprised BMI and demographic covariates, Model 2 added BOE to the covariates in model 1, and Model 3 added BOPH to Model 2. We imputed missing data for each model and repeated analyses with the larger sample sizes. To determine the extent to which random variability was responsible for the ability of the demographic variables and biomarkers to predict biospecimen availability, we randomly permuted the variable indicating the availability of biospecimens and determined a 95 % confidence interval for the percent reduction in the variance of this randomly permuted variable attributable to the covariates.

Plasma biospecimens were randomly selected (women and men, aged 35–49 years, with BMI < 25 kg/m^2^, both current smokers and non-users) and sent to the SRI Clinical Analysis Laboratory (CAL). Six clinical laboratory assays were performed on 47 plasma samples to measure levels of glucose, aspartate and alanine aminotransferases, total bilirubin, albumin, and total cholesterol. We estimated the correlation between SRI CAL plasma and original CRO serum analyte values.

PBMCs randomly selected from TES participants (*N* = 30, ~1 % of participants with available biospecimens) from defined strata [ages 35–49 and with BMI < 25 kg/m^2^] resulted in a sample that was 37 % female, 70 and 20 % self-identified White and Black, with 67 % current smokers. Initially, we performed DNA extraction from a limited number of pellets using Gentra Puregene reagents (Qiagen). To conserve biospecimen resources, we reviewed several multiple analyte protocols, and selected a protocol for simultaneous DNA and RNA extraction (NORGEN 48700 kit with Proteinase K). We modified lysis buffer amounts by available white blood cell count data and extracted ~1X10^6^ cells from each lysed pellet. DNA was sent to the Rutgers University Cell and DNA resource for genotyping with the Smokescreen® Array [6]. RNA quality (RNA integrity score, RIN) was analyzed using the Agilent 2100 BioAnalyzer using the Eukaryote Total RNA Nano assay.

Statistical analyses were performed using SAS version 9.2 (Cary, North Carolina) and STATA SE version 12.0 (Stata Corp, College Station, Texas). Except where specified, the alpha used for statistical significance was 0.05. We evaluated power to detect genetic variants for serum cotinine at genome-wide significance using Quanto [7].

## Results

### Review of the published TES literature

Scientists employed by the tobacco company sponsor have published analyses in peer-reviewed scientific journals using data from the TES pilot study [8] and the TES main study [2, 3, 9–16]. Analyses included population estimates of BOE levels for smokers and non-users [2], estimates of levels of BOPH in smokers and non-users [3], the relationships between machine-derived tar yields of cigarette products and BOE in smokers [9], models of BOPH [11], the impact of menthol-containing cigarettes on selected BOE in White and Black smokers [10], and the relationships between selected BOE and BOPH in smokers [14]. These scientists have also reported on the relationships between BOE and nicotine dependence [13] and between nicotine and carbon monoxide BOE and other factors, including smoking topographical variables [12]. These authors utilized TES data to examine the relationships between smoking mentholated cigarettes or non-mentholated cigarettes and glucuronide metabolite ratios [15], and with measures of nicotine dependence [16]. We review Roethig *et al.* [2] and Frost-Pineda *et al.* [3] here to introduce TES BOE (Additional file 2: Table S1) and BOPH (Additional file 3: Table S2).

Roethig *et al.* published estimates of BOE (Additional file 2: Table S1) in smokers and non-users and, within smokers, within different age, sex, BMI, and self-identified racial strata [2]. Mean levels of BOE were weighted by age, sex and BMI variance estimates from the U.S. Behavioral Risk Factor Surveillance System (BRFSS), an annual telephone-based behavioral survey established in 1984 [17], to produce weighted estimates of BOE reported and described by Roethig *et al.* as population estimates [2]. The BRFSS used post-stratification weighting based on United States Census data from the 1980s until 2011 [17]. Lee and Messiah criticized the application of weights extracted from a nationally representative sample to a sample for which inclusion rates at recruitment sites were not known or not reported [18]. In response, Sarkar and Liang noted that the weighted means were similar to or unchanged from unadjusted means [19]. Weighted estimates of tobacco-specific biomarkers [nicotine, cotinine and trans-3′-hydroxycotinine and their glucoronides (nicotine equivalents, NE), serum cotinine, and total 4-(methylnitrosamino)-1-(3-pyridyl)-1-butanol and glucuronide (total NNAL)] suggested that the younger participants (21–34 years) and female participants had the lowest tobacco-specific exposures, and that individuals with BMIs < 25 kg/m^2^ compared with individuals with BMIs ≥ 25 kg/m^2^ had higher serum cotinine levels and lower total NNAL levels, suggesting reduced cotinine clearance and NNK metabolism in heavier individuals [2]. Significant differences in serum cotinine by BMI similar to those reported in the TES have been previously observed in the National Health and Nutrition Examination Survey [20]. In the TES, self-identified White smokers smoked significantly more cigarettes per day and had greater NE and total NNAL exposure over 24 h, but lower NE and total NNAL exposure per cigarette, and lower serum cotinine exposure, than self-identified Black smokers [2]. It has previously been observed that White smokers smoke more cigarettes per day than Black smokers and that nicotine intake per cigarette measured by serum cotinine is higher in Black smokers than in White smokers [21, 22], which is related to significantly reduced nicotine clearance in Blacks compared to Whites [23, 24].

Frost-Pineda *et al.* [3] reported mean values for 29 BOPH (Additional file 3: Table S2) in both smokers and non-users. The BOPH represented various physiological functions: cardiovascular, endothelial, hematologic, inflammation, lipid, hepatic, renal, respiratory, metabolic, and oxidative stress [3]. The effects of multiple BOE [cigarettes per day (CPD), NE, and smoking duration] on the BOPH in current smokers versus non-users were evaluated in two stepwise regression models (model A with CPD and smoking duration, and model B with NE and smoking duration) with age, sex, BMI and self-identified race as additional independent variables [3]. The three most elevated mean BOPH in current smokers versus non-users were those reflecting oxidative stress, platelet activation and inflammation. The oxidative stress biomarker 8-epi-prostaglandin F_2α_ exhibited the largest difference between smokers and non-users (+42 %), while BMI and age, and BMI and NE, were the most important correlates in models A and B, respectively. The platelet activation biomarker 11-dehydrothromboxane B_2_ exhibited the second largest difference between smokers and non-users (+29 %), and sex and BMI, and sex and NE were the most important correlates in models A and B, respectively. The inflammation biomarker white blood cell count exhibited the third largest difference between smokers and non-users (+19 %) and BMI and self-identified race were the most important correlates in both models. Overall, BMI and sex were the first and second most common significant correlates reported by Frost-Pineda *et al.* [3].

Our search of the UCSF LTDL identified multiple documents we had received from the VTHRR, including: the Amended Final Research Protocol, dated 19 August 2002, that describes the clinical protocol, laboratory testing and biospecimen banking procedures to be conducted by the primary clinical and laboratory CRO [1]; the TES Adult Smoker Survey [25]; and the TES Adult Non-Smoker Survey [26]. We found no differences between the documents we had received from VTHRR and those available on the UCSF LTDL. We also identified summary documents that provided information on the design and analysis goals of the TES, including: a Statement of Work for data management and analysis to be conducted by the primary data analysis CRO dated 30 April 2004 [27]; a draft version of the Statistical Analysis Plan dated 1 September 2004 [28]; and a PowerPoint presentation dated 7 February 2005 that presented TES pilot results, and design and initial analyses of the TES [29]. Review of these documents enriched our understanding of the design and conduct of the study and confirmed study parameters, e.g., numbers of individuals recruited within design strata. With the assistance of a UCSF Industry Documents Digital Librarian, we identified SAS datasets available in the Philip Morris collection but these refer to an unrelated study [30].

### TES recruitment and analysis strata

The original enrollment goal of the TES [1] was 1000 smokers among four strata defined by FTC tar levels of the smoker’s usual cigarette (≤2.9, 3–6.9, 7–12.9, and ≥ 13 mg), and 1000 non-users [1]. The distribution of evaluable subjects in the five categories (504, 953, 1066, and 1062 smokers, and 1077 non-users) was significantly different from the design (Pearson *χ*^2^_4d.f._ = 159.9, *P* < 0.0001). The distribution of participants with clinical data by enrollment strata and by demographic strata is shown in Table 1.

**Table 1:** TES participants with clinical data, by recruitment variables and by previously utilized analysis strata

Analysis Variable		≤2.9 mg	3.0–6.9 mg	7.0–12.9 mg	≥13 mg	Smokers^a^	Non-users^b^
Sex	Female	293 (58.1)	648 (68.0)	604 (56.7)	514 (48.4)	2059 (57.4)	639 (59.3)
N (%)							
	Male	211 (41.9)	305 (32.0)	462 (43.3)	548 (51.6)	1526 (42.6)	438 (40.7)
Age, years	21–34	82 (16.3)	273 (28.6)	397 (37.2)	387 (36.4)	1139 (31.8)	358 (33.2)
N (%)	35–49	224 (44.4)	394 (41.3)	377 (35.4)	428 (40.3)	1423 (39.7)	358 (33.2)
	≥50	198 (39.3)	286 (30.0)	292 (27.4)	247 (23.3)	1023 (28.5)	361 (33.5)
BMI (kg/m^2^)	<25	177 (35.1)	392 (41.1)	425 (39.9)	411 (38.7)	1405 (39.2)	398 (37.0)
N (%)	≥25	327 (64.9)	561 (58.9)	641 (60.1)	651 (61.3)	2180 (60.8)	679 (63.0)
Total		504 (14.1)	953 (26.6)	1066 (29.7)	1062 (29.6)	3585 (76.9)	1077 (23.1)

^a^Participants smoking ≥ one manufactured cigarette per day during the last year. ^b^Non-users of tobacco or nicotine products for the last five years, and throughout the study

### TES demographics and smoking status

The demographic composition of TES participants with clinical data (*N* = 4662) was 57.9 % female, with mean (standard deviation, SD) age 42.1 (13.2) years and mean (SD) BMI 27.9 (6.7) kg/m^2^ (Table 2). Self-identified race distributions were 77.1 % “Caucasian or White”, 16.5 % “African American or Black”, and four other self-identified race categories comprising 6.5 % of participants. Only a small fraction of TES participants self-identified as Hispanic ethnicity (3.8 % of total participants). Most (76.9 %) TES participants were current smokers with mean (SD) CPD of 16.0 (8.9). Age, self-identified race, and education distributions differed significantly by smoking status (current smokers were significantly more likely to be older, self-identify as Black, and significantly less likely to have a college degree), while sex, BMI, and self-identified ethnicity (Hispanic versus Not Hispanic) did not differ by smoking status.

**Table 2:** TES demographics and smoking status, overall and among those with and without banked biospecimens

Characteristic	All	With	Without	*χ* ^2^ or *t*, *P*
Sex^a^				2.33, .13
Female	2698 (57.9)	1754 (57.1)	944 (59.4)	
Male	1964 (42.1)	1319 (42.9)	645 (40.6)	
Age (Years)^b^	42.1 (13.2)	42.5 (13.0)	41.2 (13.5)	3.0, .002
21–34	1497 (32.1)	927 (30.2)	570 (35.9)	16.83, .0002
35–49	1781 (38.2)	1222 (39.8)	559 (35.2)	
≥ 50	1384 (29.7)	924 (30.1)	460 (28.9)	
BMI (kg/m^2^)^c^	27.9 (6.7)	28.1 (6.8)	27.5 (6.5)	2.6, .009
< 25	1803 (38.7)	1161 (37.8)	642 (40.4)	3.05, .08
≥ 25	2859 (29.7)	1912 (30.1)	947 (59.6)	
Self-identified Race^e^				26.5, <.0001
White	3578 (77.1)	2420 (79.1)	1158 (73.2)	23.8, <.0001^d^
Black	765 (16.5)	447 (14.6)	318 (20.1)	
Other	121 (2.6)	80 (2.6)	41 (2.6)	
Multi-racial	73 (1.6)	48 (1.6)	25 (1.6)	
Native American	67 (1.4)	44 (1.4)	23 (1.5)	
Asian	24 (0.5)	12 (0.4)	12 (0.8)	
Ethnicity^f^				0.07, .79
Hispanic	178 (3.8)	119 (3.9)	59 (3.7)	
Not Hispanic	4458 (96.2)	2937 (96.1)	1521 (96.3)	
Education^g^				4.0, .26
< High School	356 (7.7)	230 (7.6)	126 (8.0)	
HS/some College	3292 (71.3)	2199 (72.2)	1093 (69.5)	
≥ Bachelors	969 (21.0)	615 (20.2)	354 (22.5)	
Smoking status				0.35, .55
Non-smoker	1077 (23.1)	718 (23.4)	359 (22.6)	
Smoker	3585 (76.9)	2355 (76.6)	1230 (77.4)	

^a^ *χ* ^2^_smoking status_ = 1.22, *P* = .27.^b^ *t*_smoking status_ = 3.2, P = .001. ^c^ *t*_smoking status_ = 1.33, *P* = .18. ^d^White and Black. ^e^All races, *χ* ^2^_smoking status_ = 5.81, P = .016. ^f^ *χ* ^2^_smoking status_ = 2.04, *P* = .15. ^g^ *χ* ^2^_smoking status_ = 223, *P* = <.0001

### TES banked biospecimen availability and smoking status

Two-thirds (66 %) of TES participants have banked biospecimens. PBMCs are the most common biospecimen type while urine is the least common (Table 3). Among participants with banked biospecimens, and among the four biospecimen types, there are no significant differences in sex, age and BMI proportions, but there are significant differences in smoking status (Table 4). Compared to participants with banked PBMC biospecimens, participants with banked urine biospecimens are significantly more likely to be smokers (*OR* = 1.26, 95 % CI 1.11–1.44, *P* = 0.0004).

**Table 3:** TES banked biospecimen aliquots, by biospecimen type

Aliquots per participant	PBMC^a^	RBC^b^	Plasma^c^	Urine^d^	Total
1 Aliquot	40	94	2914	321	
2 Aliquots	2923	905		2299	
3 Aliquots		1771			
Total N Aliquots	5886	7217	2914	4919	20936
Total N participants with Aliquots	2963	2770	2914	2620	3073

^a^From one 8.5 mL ACDA yellow-top tube processed in two vials. ^b^From one 10 mL K_2_EDTA tube processed into three vials. ^c^From one 10 mL K_2_EDTA tube of whole blood processed into two vials. ^d^Aliquots of 100 mL from a 24 h urine sample

**Table 4:** TES banked biospecimen availability, by biospecimen type, and by strata previously used for analysis

	Any	PBMC	RBC	Plasma	Urine
	3073	2963	2770	2914	2620
Smoking status					
Smoker	2355 (76.6)	2272 (76.7)	2120 (76.5)	2223 (76.3)	2112 (80.6)
Non-user	718 (23.4)	691 (23.3)	650 (23.5)	691 (23.7)	508 (19.4)
Sex					
Female	1714 (55.8)	1686 (56.9)	1582 (57.1)	1648 (56.6)	1495 (57.1)
Male	1359 (44.2)	1279 (43.1)	1188 (42.9)	1266 (43.4)	1125 (42.9)
Age (years)					
21–34	927 (30.2)	880 (29.7)	822 (29.7)	876 (30.1)	783 (29.9)
35–49	1222 (39.8)	1184 (40.0)	1109 (40.0)	1171 (40.2)	1057 (40.3)
≥ 50	924 (30.1)	899 (30.3)	839 (30.3)	867 (29.8)	780 (29.8)
BMI, kg/m^2^					
< 25	1161 (37.8)	1117 (37.7)	1046 (37.8)	1092 (37.5)	1000 (38.2)
≥ 25	1912 (62.2)	1846 (62.3)	1724 (62.2)	1822 (62.5)	1620 (61.8)

Pearson *χ* ^2^, by biospecimen type: Smoking Status, *χ* ^2^ = 19.59 (*P* = .00021); Sex, *χ* ^2^ = 0.22 (*P* = .97); Age, *χ* ^2^ = 0.44 (*P* = .99); BMI, *χ* ^2^ = 0.29 (*P* = .96)

### TES demographics

Participants with banked biospecimens are significantly older (age, continuous or categorical), have significantly increased BMI (continuous), and are more likely to self-identify as White compared to those without (Table 2). When stratified by ethnicity and race, self-identified non-Hispanic Black participants with banked biospecimens are significantly older and have significantly increased BMI than those without biospecimens [mean (SD) age 40.8 (10.7) vs 38.8 (11.1) years, *t* = 2.45, *P* = 0.0144, *N* = 755; mean (SD) BMI 30.5 (8.0) vs 28.5 (6.9) kg/m^2^, *t* = 3.58, *P* = 0.0004, *N* = 755, data not shown]. Significant differences in age and BMI among all participants and stratified by self-identified ethnicity and race are small (Cohen’s *d*-values = 0.10, 0.09, 0.18 and 0.27, respectively). Smoking duration, CPD (continuous and categorical), and usual cigarette FTC tar level (categorical) are significantly increased in those with banked biospecimens compared to those without, overall, and when stratified by self-identified race (Table 5). Significant differences are small; *d*-values for smoking duration overall, and among self-identified non-Hispanic Blacks and Whites are 0.13, 0.22 and 0.08, respectively, and *d*-values for CPD among self-identified non-Hispanic smokers, and among self-identified non-Hispanic White smokers, are 0.14 and 0.09, respectively.

**Table 5:** TES self-reported BOE, non-Hispanic current smokers by self-identified race, and by banked biospecimens

Characteristic	All	With	Without	*χ* ^2^ or *t*, *P*
Years smoked [N (%)]^a^	22.0 (12.9)	22.6 (12.8)	20.9 (13.0)	3.62, .0003
Black	18.6 (11.8)	19.7 (11.8)	17.1 (11.5)	2.69, .0074
White	22.8 (13.0)	23.1 (13.0)	22.0 (13.2)	2.05, .0404
CPD [N (%)]^b^	16.3 (8.9)	16.7 (8.9)	15.5 (8.9)	3.60, .0003
Black	11.3 (6.3)	11.4 (6.4)	11.1 (6.1)	0.61, .5393
White	17.4 (9.1)	17.6 (9.0)	16.8 (9.2)	2.13, .0329
CPD^c^ 1–10	952(28.8)	579 (26.6)	373 (33.1)	17.7, .0005
11–20	1487 (45.0)	996 (45.8)	491 (43.6)	
21–30	643 (19.5)	453 (20.8)	190 (16.9)	
≥ 31	221 (6.7)	148 (6.8)	73 (6.5)	
Black 1–10	327 (54.0)	179 (52.7)	148 (55.6)	0.705, .8720
11–20	227 (37.5)	130 (38.2)	97 (36.5)	
21–30	43 (7.1)	26 (7.7)	17 (6.4)	
≥ 31	9 (1.5)	5 (1.5)	4 (1.5)	
White 1–10	625 (23.2)	400 (21.8)	225 (26.1)	7.701, .0526
11–20	1260 (46.7)	866 (47.2)	394 (45.8)	
21–30	600 (22.3)	427 (23.3)	173 (20.1)	
≥ 31	212 (7.9)	143 (7.8)	69 (8.0)	
FTC tar, mg^d^	9.16 (5.4)	9.18 (5.3)	9.13 (5.6)	0.23, .8187
Black	10.9 (6.9)	10.7 (6.9)	11.2 (6.9)	–0.74, .4622
White	8.8 (5.0)	8.9 (4.9)	8.5 (5.0)	1.86, .0624
≤ 2.9	472 (14.3)	314 (14.4)	158 (14.0)	13.28, .0041
3–6.9	887 (26.8)	551 (25.3)	336 (29.8)	
7–12.9	985 (29.8)	689 (31.6)	296 (26.2)	
≥ 13	965 (29.2)	627 (28.8)	338 (30.0)	
Black ≤ 2.9	135 (22.3)	81 (23.8)	54 (20.3)	1.35, .7183
3–6.9	76 (12.5)	40 (11.8)	36 (13.5)	
7–12.9	74 (12.2)	40 (11.8)	34 (12.8)	
≥ 13	321 (53.0)	179 (52.7)	142 (53.4)	
White ≤ 2.9	337 (12.5)	233 (12.7)	104 (12.1)	14.62, .0022
3–6.9	811 (30.0)	511 (27.8)	300 (34.8)	
7–12.9	911 (33.7)	649 (35.3)	262 (30.4)	
≥ 13	644 (23.8)	448 (24.3)	196 (22.7)	

^a^ *N* = 3274. ^b^Cigarette butts returned, 24 h, *N* = 3303. ^c^FTND coding (1–10 = 0, 11–20 = 1, 21–30 = 2, >30 = 3). ^d^ *N* = 3309

### TES BOE

Most tobacco-specific (NE, serum cotinine and total NNAL) and non-specific BOE are significantly higher in smokers with banked biospecimens than in smokers without, except for serum cotinine, 4-ABP and MHBMA (Table 6). Metabolites of acreolein and 1,3 butadiene are significantly greater in non-users with banked biospecimens than in non-users without. All statistically significant differences in BOE by banked biospecimen availability have small effect sizes, ranging from 0.10 to 0.24. When stratified by self-identified ethnicity and race, more BOE differ significantly by biospecimen availability among non-Hispanic Whites than among non-Hispanic Blacks (Tables 7 and 8). The effect sizes of the two BOE differences in self-identified non-Hispanic Black smokers are small, and the effect size of the one BOE difference in self-identified non-Hispanic Black non-users is a medium effect size (*d* = 0.47). Among self-identified non-Hispanic White smokers, NE, total NNAL, carboxyhemoglobin and an acreolin metabolite, and among self-identified non-Hispanic White non-users, a 1,3 butadiene metabolite, exhibit significant differences. All these significant differences are of small effect size.

**Table 6:** TES laboratory-based BOE^a^ among smokers and non-users, and among those with and without banked biospecimens

	All	With	Without	*t*	*P*
*Among smokers*					
NE per cig^b^	0.89 (0.7)	0.91 (0.7)	0.86 (0.7)	2.33	.020
NE mg/24 hr^c^	12.78 (7.9)	13.3 (7.9)	11.76 (7.8)	5.48	<.0001
Serum cotinine ng/ml^d^	188.9 (103.4)	190.0 (103.1)	186.7 (147.7)	0.9	.37
Total NNAL ng/cig^e^	29.4 (23.7)	30.3 (23.9)	27.8 (23.1)	3.03	.0025
Total NNAL ng/24 hr^f^	425.2 (303.9)	445.5 (308.2)	385.7 (291.5)	5.67	<.0001
COHb % saturation^g^	5.26 (2.3)	5.35 (2.3)	5.09 (2.3)	3.25	.0012
1-OHP ng/24 hr^h^	259.8 (345)	268.3 (343)	243.3 (349)	2.32	.02
3-HPMA ug/24 hr^i^	1941.9 (1326)	2017.6 (1302)	1796.6 (1361)	4.73	<.0001
4-ABP pg/g Hb^j^	43.5 (53.6)	44.2 (56.8)	42.3 (47.1)	0.96	.34
MHBMA ug/24 hr^k^	3.52 (3.3)	3.60 (3.3)	3.38 (3.4)	1.87	.062
DHBMA ug/24 hr^l^	530.1 (276.3)	541.1 (255.7)	508.9 (311.2)	3.11	.0019
*Among non-smokers*					
COHb % saturation^m^	1.46 (0.5)	1.46 (0.6)	1.45 (0.5)	0.08	.94
3-HPMA ug/24 hr^n^	461.7 (532)	484.3 (571)	416.3 (441.0)	2.14	.033
4-ABP pg/g Hb^o^	14.5 (70.8)	13.2 (71.5)	17.0 (69.6)	−0.68	.5
MHBMA ug/24 hr^p^	0.49 (1.0)	0.48 (0.8)	0.50 (1.3)	−0.16	.88
DHBMA ug/24 hr^q^	385.6 (172.5)	398.8 (182.3)	359.1 (147.7)	3.82	<.0001

^a^Definitions (and parent compounds) of BOE from Additional file 2: Table S1: *NE* Nicotine Equivalents (Nicotine); *Total NNAL* Total 4-(methylnitrosamino)-1-(3-pyridyl)-1-butanol, and its glucuronide (4-(methylnitrosamino)-1-(3-pyridyl)-1-butanone); *COHb* carboxyhemoglobin (carbon monoxide); *1-OHP* Total 1-hydroxypyrene (polycyclic aromatic hydrocarbons); *3-HPMA* 3-hydroxy-propylmercapturic acid (acreolein); *4-ABP* 4-aminobiphenyl hemoglobin (Hb) adducts (4-aminobiphenyl); *MHBMA* monohydroxyl-butenylmercapturic acid (1,3 butadiene); DHBMA = dihydroxy-butyl-mercapturic acid (1,3 butadiene)

^b^ *N* = 3529, *d* = 0.10. ^c^ *N* = 3535, *d* = 0.18. ^d^ *N* = 3469. ^e^ *N* = 3529, *d* = 0.11. ^f^ *N* = 3535, *d* = 0.20. ^g^ *N* = 3558, *d* = 0.11. ^h^ *N* = 3554, *d* = 0.07. ^i^ *N* = 3556, *d* = 0.17. ^j^ *N* = 2801. ^k^ *N* = 3415. ^l^ *N* = 3558, *d* = 0.11. ^m^ *N* = 1069. ^n^ *N* = 1058, *d* = 0.13. ^o^ *N* = 723. ^p^ *N* = 629. ^q^ *N* = 1074, *d* = 0.24

**Table 7:** TES Non-Hispanic Black laboratory-based BOE by smoking status, and by banked biospecimen availability

	All	With	Without	*t*	*P*
*Among smokers*					
NE per cig^a^	1.04 (0.8)	1.05 (0.8)	1.02 (0.9)	0.49	.6254
NE mg/24 hr^a^	10.3 (6.7)	10.6 (6.6)	9.82 (6.7)	1.46	.1449
Serum cotinine ng/ml^b^	205.3 (113)	206.8 (114.5)	203.2 (111.2)	0.37	.7081
Total NNAL ng/cig^c^	33.4 (27.5)	34.8 (28.0)	31.5 (26.8)	1.47	.1414
Total NNAL ng/24 hr^3^	331.8 (233.9)	349.7 (236.9)	308.6 (228.3)	2.13	.0336
COHb % saturation^d^	4.74 (2.1)	4.75 (2.1)	4.74 (2.1)	0.08	.9355
1-OHP ng/24 hr^e^	326.4 (421)	333.8 (379.8)	316.9 (469)	0.47	.6352
3-HPMA ug/24 hr^f^	1605 (1083)	1670 (1005)	1521 (1172)	1.64	.1012
4-ABP pg/g Hb^g^	39.6 (28.2)	40.1 (29.8)	39.0 (26.6)	0.41	.6831
MHBMA ug/24 hr^h^	2.65 (2.8)	2.9 (3.2)	2.3 (2.1)	2.75	.0062
DHBMA ug/24 hr^i^	495.5 (270.2)	511.7 (276.9)	474.4 (260.4)	1.67	.0946
*Among non-smokers*					
COHb % saturation^j^	1.5 (0.7)	1.49 (0.7)	1.52 (0.6)	−0.24	.8116
3-HPMA ug/24 hr^k^	433.5 (379.8)	458.8 (430.1)	380.3 (237.3)	1.43	.1546
4-ABP pg/g Hb^l^	12.9 (16.6)	14.7 (19.7)	9.24 (5.7)	1.96	.054
MHBMA ug/24 hr^m^	0.57 (0.9)	0.64 (1.1)	0.42 (0.6)	1.41	.1619
DHBMA ug/24 hr^n^	401.3 (177)	426.3 (188.3)	349.2 (138.8)	2.83	.0054

^a^ *N* = 574. ^b^ *N* = 576. ^c^ *N* = 593. ^d^ *N* = 603. ^e^ *N* = 603. ^f^ *N* = 601. ^g^ *N* = 478. ^h^ *N* = 569. ^i^ *N* = 598. ^j^ *N* = 148. ^k^ *N* = 149. ^l^ *N* = 87. ^m^ *N* = 103. ^n^ *N* = 151

**Table 8:** TES non-Hispanic White laboratory-based BOE, by smoking status, and by banked biospecimen availability

	All	With	Without	*t*	*P*
*Among smokers*					
NE per cig^a^	0.86 (0.6)	0.88 (0.6)	0.82 (0.6)	2.4	.0166
NE mg/24 hr^b^	13.5 (8.1)	14.0 (8.1)	12.6 (8.1)	4.01	<.0001
Serum cotinine ng/ml^c^	187.1 (101.2)	188.1 (100.6)	184.9 (102.6)	0.75	.4545
Total NNAL ng/cig^d^	28.4 (22.4)	29.2 (22.4)	26.7 (22.1)	2.7	.0069
Total NNAL ng/24 hr^e^	450.9 (314.2)	467.1 (315.6)	416.2 (308.5)	3.91	<.0001
COHb % saturation^f^	5.43 (2.3)	5.49 (2.3)	5.29 (2.4)	2.12	.0343
1-OHP ng/24 hr^g^	300.4 (362.3)	307.1 (361.0)	286.3 (364.7)	1.38	.1669
3-HPMA ug/24 hr^h^	2048 (1368)	2100 (1337)	1936 (1427)	2.89	.0039
4-ABP pg/g Hb^i^	45.2 (59.6)	45.7 (62.3)	44.1 (53.6)	0.6	.5475
MHBMA ug/24 hr^j^	3.78 (3.4)	3.76 (3.2)	3.82 (3.7)	−0.39	.6985
DHBMA ug/24 hr^k^	541.6 (281.2)	548.3 (252.9)	527.4 (333.4)	1.63	.1029
*Among non-smokers*					
COHb % saturation^l^	1.44 (0.5)	1.44 (0.5)	1.44 (0.4)	0.07	.9449
3-HPMA ug/24 hr^m^	463 (557.3)	480.2 (595)	429.3 (474.6)	1.35	.1791
4-ABP pg/g Hb^n^	15.1 (78.1)	13.1 (79.3)	18.7 (76.0)	−0.83	.4085
MHBMA ug/24 hr^o^	0.47 (1.0)	0.44 (0.7)	0.53 (1.5)	−0.71	.4782
DHBMA ug/24 hr^p^	386.1 (170.6)	397.1 (180.1)	364.7 (148.5)	2.8	.0053

^a^ *N* = 2680. ^b^ *N* = 2686. ^c^ *N* = 2635. ^d^ *N* = 2667. ^e^ *N* = 2673. ^f^ *N* = 2680. ^g^ *N* = 2683. ^h^ *N* = 2681. ^i^ *N* = 2119. ^j^ *N* = 2590. ^k^ *N* = 2684. ^l^ *N* = 846. ^m^ *N* = 837. ^n^ *N* = 589. ^o^ *N* = 484. ^p^ *N* = 848

### TES BOPH

The distribution of BOPH by banked biospecimen availability is shown in Table 9. Six of 29 BOPH measures have nominally significantly higher levels in TES participants with available banked biospecimens versus those without, while the respiratory function measure FVC and hemoglobin remain significantly different after false discovery rate correction (*q*-values = 0.0128 and 0.0496, respectively) [31]. After excluding individuals with implausible FEV_1_ values < 35 % or > 125 % of predicted, as suggested by Frost-Pineda *et al.* [3], and then stratifying by self-identified ethnicity and race, and then by smoking status, we observed that self-identified non-Hispanic White smokers with banked biospecimens have significantly increased FVC compared to those without [93.9 (23.2) vs 90.8 (17.9), *t =* 3.81, *P =* 0.0001, *N* = 2584]. The statistically significant increase in % predicted FVC among self-identified non-Hispanic White smokers with available biospecimens is unexpected because multiple BOE are significantly increased in self-identified non-Hispanic White smokers with banked biospecimens and lung function is expected to be reduced in individuals with increased measures of exposure. Evidence for the influence of current smoking on longitudinal decline in FEV_1_ and FVC suggests that current smoking influences longitudinal FEV_1_ decline more than FVC [32], though this would not explain an increase in FVC. We constructed another regression model including education and household income, but these potential confounders [33] had no effect on the observed differences in FVC within non-Hispanic White smokers (data not shown). Further analyses of lung function measures and other variables in the TES may identify possible explanatory factors or confounders. After stratifying by self-identified race and ethnicity, and then by smoking status, we observed that self-identified non-Hispanic White non-users exhibit a significant difference in hemoglobin by banked biospecimen availability [14.50 (1.42) vs 14.31 (1.25), *t =* 1.84, *P =* 0.033, *N* = 828]. Statistically significant differences in FVC and hemoglobin in these strata are small (*d*-values are 0.15 and 0.14, respectively).

**Table 9:** TES participant BOPH, by banked biospecimen availability

Analyte	With	Without	N	*t*	*P*
8-*epi*-prostaglandin-F_2α_^a^	1745 (1000)	1657 (1056)	4557	2.74	.006
Total bilirubin^b^	0.48 (0.3)	0.49 (0.3)	4420	−0.92	.36
Hematocrit (%)	43.2 (4.1)	42.9 (4.1)	4560	2.61	.01
Hemoglobin^c^	14.7 (1.52)	14.6 (1.50)	4572	2.74	.003
Platelets (10^3^/uL)	275.2 (70.2)	277.9 (71.0)	4527	−1.25	.21
WBC (10^3^/uL)	7.68 (2.3)	7.61 (2.2)	4572	0.99	.32
Microalbumin (mg/24 h)	43.7 (338.3)	31.6 (188.4)	3235	1.31	.19
11-dehydrothromboxane-B_2_^1^	1343.0 (931)	1277.2 (1027)	4286	2.05	.04
hs-CRP (mg/L)	4.45 (7.0)	4.25 (6.4)	4433	0.92	.36
Fibrinogen^b^	323.8 (75.8)	320.1 (77.7)	4498	1.56	.12
von Willebrand Factor (%)	102.8 (46.1)	100.9 (48.6)	4601	1.26	.21
Serum creatinine^b^	0.82 (0.2)	0.81 (0.2)	4640	0.32	.75
Blood urea nitrogen^b^	13.5 (4.4)	13.1 (4.5)	4640	2.39	.02
Total cholesterol^b^	196.8 (40.9)	194.6 (42.3)	4639	1.68	.09
HDL^b^	52.2 (16.3)	52.6 (16.2)	4590	−0.95	.34
LDL^b^	115.3 (35.0)	113.5 (37.4)	4428	1.60	.11
Triglycerides^b^	153.9 (125)	148.9 (125)	4639	1.29	.19
Alkaline phosphatase^d^	74.9 (25.3)	74.9 (26.1)	4637	−0.02	.99
Alanine aminotransferase^d^	26.1 (24.7)	26.5 (24.1)	4617	−0.45	.65
Aspartate aminotransferase^d^	24.8 (19.0)	25.4 (21.1)	4561	−0.96	.34
Lactate dehydrogenase^d^	154.8 (32.8)	153.1 (32.2)	4482	1.63	.10
FEV_1_ (% of predicted)	85.1 (21.7)	84.7 (20.9)	4539	0.48	.63
FVC (% of predicted)	94.4 (25.1)	91.8 (22.0)	4541	3.52	.0004
Serum albumin^c^	4.31 (0.4)	4.30 (0.3)	4629	0.27	.79
Serum glucose^b^	96.8 (30.7)	97.1 (29.8)	4626	−0.29	.77
Uric acid^b^	5.28 (1.5)	5.26 (1.4)	4640	0.40	.69
Diastolic BP (mmHg), Visit 1	77.0 (10.7)	76.5 (10.3)	4659	1.26	.21
Diastolic BP (mmHg), Visit 2	76.5 (10.3)	76.5 (10.2)	4660	−0.05	.96
Systolic BP (mmHg), Visit 1	123.5 (16.6)	122.6 (16.0)	4659	1.78	.07
Systolic BP (mmHg), Visit 2	121.5 (15.9)	120.8 (15.5)	4660	1.32	.19
Heart rate (bpm), Visit 1	72.7 (10.2)	72.8 (10.2)	4656	−0.12	.90
Heart rate (bpm), Visit 2	73.4 (10.3)	72.9 (10.4)	4659	1.44	.15

^a^ng/24 hrs. ^b^mg/dL. ^c^g/dL. ^d^U/L

### TES participant usual cigarette brand

Information on participant’s usual cigarette brand is available from 606 and 1336 self-identified non-Hispanic Black and non-Hispanic White smokers, respectively. The top 20 brands account for 66.0 and 49.4 % of the brand information available from self-identified non-Hispanic Black and non-Hispanic White smokers, respectively (Table 10). Usual cigarette brand distributions do not differ significantly among self-identified non-Hispanic Black or among non-Hispanic White smokers by the presence or absence of banked biospecimens (Table 10).

**Table 10:** TES participant usual cigarette brand, by self-identified race/ethnicity and by banked biospecimen availability

Usual cigarette brand identification	All	With [N, %]		Without [N, %]	
	Self-identified non-Hispanic Black smokers^a^				
Newport KFMHP 263101	101	51	50.5	50	49.5
Newport 100’s 100FMHP	64	35	54.7	29	43.5
Carlton menthol KFMSP	26	17	65.4	9	34.6
Newport KFMSP 278012	23	12	52.2	11	47.8
Carlton 100’s 100FHP	20	10	50.0	10	50.0
Carlton 100’s 100FSP	18	9	50.0	9	50.0
Newport 100’s 100FMSP	17	7	41.2	10	58.8
Carlton 100’s menthol 100FMSP	16	12	75.0	4	25.0
Carlton 100’s menthol 100 FMSP	15	9	60.0	6	40.0
Carlton KFSP	12	8	66.7	4	33.3
Benson & Hedges 100’s menthol 100FMSP	11	9	81.8	2	18.2
Kool filter kings KFMHP	11	7	63.6	4	36.4
Merit ultra lights KFHP	11	4	36.4	7	63.6
Kool filter king KFMSP	9	6	66.7	3	33.3
Kool super longs 100’s 100 FMHP	9	4	44.4	5	55.6
Kool super longs 100’s 100FMSP	9	5	55.6	4	44.4
Merit ultima 100’s 100FHP	8	5	62.5	3	37.5
Now 100 s menthol 100 FMSP	8	5	62.5	3	37.5
Carlton KFHP	6	4	66.7	2	33.3
Doral full flavor 100’s menthol 100FMSP	6	3	50.0	3	50.0
Total	400	222	55.5	178	44.5
	Self-identified non-Hispanic White smokers^b^				
Marlboro lights KFHP	227	160	70.5	67	29.5
Marlboro ultra lights KFHP	186	124	66.7	62	33.3
Marlboro KFHP	147	103	70.1	44	29.9
Marlboro ultra lights 100’s 100FHP	106	70	66.0	36	34.0
Marlboro lights 100’s 100FHP	90	68	75.6	22	24.4
Camel Turkish lights KFHP	81	55	67.9	26	32.1
Carlton 100’s 100FHP	67	46	68.7	21	31.3
Marlboro 100’s 100FHP	61	42	68.9	19	31.1
Carlton 100’s 100FSP	47	28	59.6	19	40.3
Camel filters KFHP	41	26	63.4	15	36.6
Carlton KFSP	35	26	74.3	9	25.7
Newport KFMHP 263101	33	25	75.8	8	24.2
Now 100 s 100FSP	31	24	77.4	7	22.6
Doral ultra lights 100’s 100FHP	30	19	63.3	11	36.7
Virginia slims ultra lights menthol 100FMHP	29	19	65.5	10	34.5
Benson & Hedges deluxe ultra lights 100’s 100FHP	26	12	46.2	14	53.9
Now 100 s menthol 100FMSP	26	21	80.8	5	19.2
Carlton 100’s menthol 100FMSP	25	20	80.0	5	20.0
Marlboro KFSP	24	17	70.8	7	29.2
Virginia slims ultra lights 100FHP	24	18	75.0	6	25.0
Total	1336	923	69.1	413	30.9

^a^χ^2^_19d.f._ = 13.85, *P* = .79. ^b^ *χ* ^2^_19d.f._ = 18.34, *P* = .50

### Modeling banked biospecimen availability by demographics, BOE and BOPH

Sample sizes among self-identified non-Hispanic Black and White smokers with complete data and with imputed data for the progressively more complex models were 3236 and 3318 (2.5 % of participants had missing data in Model 1), 2317 and 3318 (30.2 % of participants had missing data in Model 2), and 1090 and 3053 (64.3 % had missing data in Model 3), respectively. However, while a large fraction of the population was missing one or more variable values, on average they were only missing a single value out of a large number of independent variables. The number of missing values that were imputed was relatively small; 0.3 % of all values required imputation in Model 1, 1.9 % in Model 2, and 2.5 % in Model 3. Significant demographic variables, BOE and BOPH in progressively more complex multivariate models of banked biospecimen availability with imputed data were: Model 1) BMI, self-identified race, age and age squared; Model 2) self-identified race, age, age squared, and NE/24 h; and Model 3) self-identified race, age, age squared, NE/24 h, serum cotinine, MHBMA and FVC (Table 11). The mean (SD) predicted probabilities of banked biospecimen availability, in progressively more complex multivariate models without and with imputed data are: Model 1) 0.647 (0.069) and 0.665 (0.060); Model 2) 0.643 (0.077) and 0.668 (0.070); and Model 3) 0.625 (0.102) and 0.669 (0.095). Explanatory power estimates (r^2^) of the anthropometric, demographic, BOE and BOPH variables in progressively more complex multivariate models with imputed data among self-identified non-Hispanic Black and White smokers to predict banked biospecimen availability are 0.018, 0.024, and 0.037, respectively. In permutation analyses of self-identified non-Hispanic Black and White smokers with imputed data in Model 3, the mean (95 % confidence interval) r^2^ was 0.020 (0.016 - 0.023) suggesting that about half of the explanatory power of variables is due to random variability (0.020/0.037 = 0.54).

**Table 11:** Multivariate model of banked biospecimen availability, self-identified non-Hispanic black and non-Hispanic white smokers

	*β*	SE	*t*	*P*	FMI^a^
Sex	0.191	0.123	1.550	0.121	0.009
BMI^b^	0.015	0.008	1.860	0.063	0.010
Self-identified race	−0.598	0.125	−4.780	0.000	0.006
Education (HS/some College)	0.001	0.146	0.010	0.993	0.011
Education (≥Bachelors)	−0.129	0.173	−0.740	0.457	0.010
Age	0.057	0.020	2.800	0.005	0.003
Age squared	−0.001	0.000	−2.930	0.003	0.003
Smoking duration	0.007	0.006	1.280	0.201	0.031
11–20 CPD	0.101	0.114	0.890	0.374	0.009
21–30 CPD	0.143	0.162	0.880	0.379	0.010
>30 CPD	0.215	0.243	0.880	0.377	0.020
CPD^b^	0.001	0.009	0.070	0.944	0.011
FTC tar	−0.002	0.008	−0.300	0.767	0.004
Nicotine equivalents/cigarette	−0.085	0.162	−0.530	0.599	0.042
Nicotine equivalents/24 h	0.034	0.015	2.350	0.019	0.028
Serum cotinine	−0.001	0.001	−2.070	0.038	0.027
Total NNAL/cigarette	0.006	0.005	1.220	0.224	0.034
Total NNAL/24 h	0.000	0.000	−0.190	0.849	0.025
Carboxyhemoglobin	−0.019	0.027	−0.700	0.486	0.007
1-OHP	0.000	0.000	0.000	0.999	0.005
3-HPMA	0.000	0.000	0.010	0.990	0.010
4-ABP	0.001	0.001	0.840	0.400	0.317
MHBMA	−0.030	0.015	−1.980	0.048	0.069
DHBMA	0.000	0.000	−0.820	0.410	0.006
8-*epi*-prostaglandin-F_2α_^b^	0.000	0.000	−0.630	0.527	0.010
Total bilirubin	−0.147	0.177	−0.830	0.406	0.051
Hematocrit	0.018	0.026	0.680	0.495	0.027
Hemoglobin	−0.071	0.077	−0.930	0.351	0.024
Platelets	−0.001	0.001	−1.280	0.200	0.051
WBC	−0.007	0.020	−0.370	0.715	0.026
Microalbumin	0.000	0.000	0.960	0.341	0.409
11-dehydrothromboxane-B_2_	0.000	0.000	0.720	0.472	0.108
hs-CRP	0.007	0.007	1.020	0.306	0.072
Fibrinogen	0.000	0.001	0.560	0.575	0.052
von Willebrand factor	0.000	0.001	0.260	0.798	0.019
Serum creatinine	0.220	0.259	0.850	0.396	0.021
Blood urea nitrogen	−0.004	0.011	−0.360	0.720	0.005
Total cholesterol	0.021	0.138	0.160	0.877	0.030
HDL	−0.019	0.138	−0.140	0.890	0.030
LDL	−0.022	0.138	−0.160	0.872	0.030
Triglycerides	−0.004	0.028	−0.150	0.882	0.031
Alkaline phosphatase	−0.001	0.002	−0.840	0.401	0.009
Alanine aminotransferase	0.001	0.003	0.380	0.700	0.012
Aspartate aminotransferase	−0.006	0.004	−1.580	0.113	0.022
Lactate dehydrogenase	0.003	0.001	1.880	0.060	0.041
FEV_1_ excluding extreme values^b^	−0.003	0.003	−0.930	0.352	0.002
FVC excluding extreme values^b^	0.010	0.003	4.030	0.000	0.003
Serum albumin	0.178	0.141	1.260	0.206	0.022
Serum glucose	−0.003	0.001	−1.850	0.065	0.019
Uric acid	−0.027	0.037	−0.730	0.464	0.004
Diastolic BP, Visit 1	0.005	0.006	0.830	0.405	0.003
Diastolic BP, Visit 2	−0.005	0.006	−0.760	0.446	0.002
Systolic BP, Visit 1	0.000	0.004	0.100	0.919	0.003
Systolic BP, Visit 2	0.003	0.004	0.680	0.498	0.002
Heart rate, Visit 1	−0.007	0.005	−1.500	0.134	0.003
Heart rate, Visit 2	0.003	0.005	0.580	0.563	0.003
Constant	−2.243	0.988	−2.270	0.023	0.006

^a^Proportion of variability in the SE due to multiple imputation. ^b^lmputation truncated at observed values

**Fig. 1:**
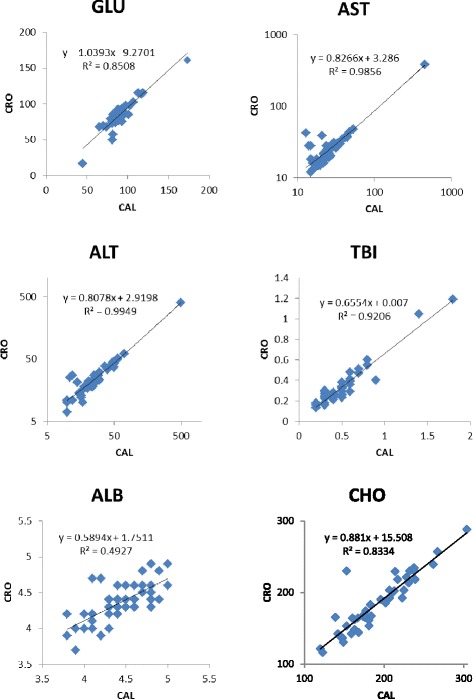
Comparison of six circulating analytes in TES plasma (CAL) and serum (CRO)

Correlations of clinical chemistry results in 47 plasma samples from the SRI CAL (2013) and those from serum reported by the CRO (2002–2003) were high and statistically significant [glucose (0.922), aspartate aminotransferase (0.993), alanine aminotransferase (0.997), total bilirubin (0.960), albumin (0.702), and total cholesterol (0.913), all p-values < 0.001] (Table 12 and Fig. 1). The lower correlation for blood albumin may be due to the two different matrices, the increased variance of some albumin clinical chemistry analysis methods [34], or the use of different methods in the clinical analyzers in the two different clinical chemistry laboratories.

**Table 12:** Comparison of six circulating analytes in TES participant plasma and serum

SRI CAL analyte values, plasma						CRO analyte values, serum					
GLU^a^	AST^b^	ALT^c^	TBI^d^	ALB^e^	CHO^f^	GLU	AST	ALT	TBI	ALB	CHO
89	455	495	1.8	4.4	223	82	381	403	1.19	4.2	192
117	20	18	1.4	4.0	213	114	17	18	1.05	4.0	202
173	22	42	0.4	4.7	238	161	21	37	0.28	4.5	234
84	45	50	0.5	4.2	184	83	37	37	0.23	3.9	167
86	30	29	0.4	4.3	202	83	31	27	0.28	4.4	185
119	27	22	0.6	4.6	142	115	20	19	0.41	4.6	142
65	22	22	0.5	4.5	207	68	22	21	0.38	4.4	203
85	31	36	0.6	4.5	237	85	27	38	0.36	4.6	234
70	32	45	0.2	4.4	163	69	26	39	0.18	4.4	148
113	16	10	0.2	4.1	176	115	13	11	0.16	4.0	165
85	22	22	0.8	4.7	304	74	22	20	0.60	4.8	288
80	38	57	0.3	4.7	160	79	34	52	0.24	4.6	164
94	17	12	0.4	4.6	148	93	14	11	0.27	4.3	136
94	24	21	0.3	5.0	231	91	28	22	0.22	4.9	213
93	18	25	0.3	4.0	238	92	17	27	0.27	4.2	230
103	21	27	0.3	4.1	173	98	18	23	0.29	4.2	167
93	27	28	0.8	4.3	145	93	20	24	0.55	4.0	138
79	23	22	0.6	4.2	217	79	19	19	0.48	4.7	218
83	19	16	0.5	4.6	174	82	15	13	0.33	4.3	166
93	22	15	0.4	4.8	149	86	16	14	0.24	4.3	130
85	26	24	0.6	5.0	262	79	19	18	0.29	4.6	239
93	25	30	0.4	4.4	176	75	21	31	0.23	4.2	167
81	15	14	0.7	4.8	214	50	28	21	0.47	4.9	229
91	18	10	0.3	4.4	211	81	18	10	0.18	4.3	200
90	26	25	0.5	4.6	177	84	22	21	0.33	4.4	174
92	36	56	0.4	3.9	228	91	30	50	0.21	4.0	221
84	13	11	0.4	4.1	232	79	42	25	0.26	4.2	230
90	16	16		4.8	123	86	17	13	0.15	4.6	116
82	21	10	0.3	3.8	196	57	39	7	0.25	4.2	190
82	25	20	0.5	3.9	159	85	20	17	0.29	3.7	142
74	48	29	0.9	4.8	181	68	44	24	0.40	4.4	161
107	42	70	0.7	4.9	200	102	37	60	0.51	4.8	187
88	22	25	0.5	3.8	167	85	21	20	0.34	3.9	164
90	25	27	0.5	4.4	183	88	25	27	0.34	4.6	183
98	27	18	0.4	4.5	207	98	25	18	0.27	4.3	192
94	44	47	0.2	4.1	163	89	39	44	0.16	4.1	162
89	20	17	0.3	4.3	139	92	15	10	0.30	4.3	165
93	32	51	0.4	4.8	225	82	26	42	0.24	4.5	203
81	15	16	0.5	4.3	181	75	12	12	0.26	4.2	153
81	53	47	0.6	4.5	153	73	48	41	0.39	4.3	153
45	15	17	0.3	4.3	120	17	18	16	0.16	4.4	121
89	23	23	0.5	4.9	168	77	18	18	0.32	4.4	144
86	33	30	0.3	4.4	267	82	30	23	0.16	4.4	257
101	16	16	0.2	4.4	239	85	16	15	0.13	4.3	218
90	33	40	0.4	4.3	177	83	27	34	0.27	4.3	166
84	26	24	0.4	4.3	175	80	27	23	0.25	4.3	169
88	14	12	0.2	4.1	153	92	28	27	0.18	4.7	230

^a^Glucose (mg/dL). ^b^Aspartate aminotransferase (U/L). ^c^Alanine aminotransferase (U/L). ^d^Total bilirubin (mg/dL). ^e^Albumin (g/dL). ^f^Total cholesterol (mg/dL)

Mean (SD) DNA and RNA from ~1 M cells was 4.63 (1.63) ug, and 2.10 (0.62) ug, respectively. We sent four DNA samples from Gentra Puregene extraction and 27 DNA samples from NORGEN extraction for Smokescreen Array genotyping at the Rutgers University Infinite Biologics facility. All DNA samples had genotype completion rates ≥ 97.5 % and passed the 97 % rate threshold; the mean genotype completion rate was 99.4 %. Mean (SD) RNA Integrity (RIN) scores from 28 RNA samples analyzed were 6.4 (2.2); 68 % of RIN scores were ≥ 6.0, a standard used in RNA sequence analysis [35]. There were no significant differences in sex, race, smoking status, or total nicotine equivalents between RNA samples with RIN ≥ 6.0 and < 6.0 (all *p*-values > 0.12). Thus, from PBMC pellets frozen at ultralow temperatures for over a decade, DNA quality and genotyping results were excellent, while RNA quality was good, but requires evaluation using transcriptome-wide methods.

Finally, we assessed statistical power to detect *a priori* genetic loci of interest from an example of a large-scale (1000 s) candidate gene association scan, and an example from a locus nominated by genome-wide association scans (GWAS), with genome-wide significance (GWS) as the statistical threshold. For self-identified non-Hispanic Black current smokers, we selected rs11187065 as an example, identified in the insulin-degrading enzyme gene as the gene-centric SNP most significantly associated with serum cotinine in the Coronary Artery Risk Development in Young Adults (CARDIA) study by Hamidovic *et al.* [36]. The influence of rs11187065 on serum cotinine was substantial with a *β* of −85.1 ng/ml, with mean (SE) of 236.5 (8.1) ng/ml from 365 African American smokers [36]. Mean (SD) CPD in the CARDIA sample was 10.5 (7.4), similar to that of the TES (Table 4). Using the sample size of self-identified non-Hispanic Black smokers with banked PBMCs in the TES (*N* = 340), there is 57 % power to detect the locus at genome-wide significance (and 83 % power to detect this locus at the original study’s Bonferroni adjustment level of 2.3 × 10^−6^) using an additive model, a one-sided test, the mean (SD) of serum cotinine among self-identified non-Hispanic Black smokers (Table 6), the rs11187065 minor allele frequency of 0.083 in the HapMap [37] African Americans in the Southwest sample, and the effect size from Hamidovic *et al*. For assessing power to detect *a priori* loci of interest among self-identified non-Hispanic White current smokers, we selected rs1051730 in the nicotinic acetylcholine receptor (nAChR) subunit gene cluster on chromosome 15q25.1, associated with smoking intensity and related phenotypes [38], including cotinine level [39], as an example. In an analysis of 2932 smokers with serum or plasma cotinine estimates, Munafo *et al.* estimated that each minor allele contributed to a mean increase in the unadjusted level of cotinine in European ancestry samples of 138.72 nmol/L [(95 % CI) 97.91 - 179.53 nmol/L, *P* = 2.7 × 10^-^^11^] [39], or 24.42 ng/mL, although this was reduced 18 % upon adjustment for self-reported CPD. Using the sample size of self-identified non-Hispanic White smokers with banked PBMCs in the TES (*N* = 1840), there is 70–96 % power to detect this locus at a genome wide significance level (5 × 10^−8^) using an additive model, a one-sided test, the mean (SD) of serum cotinine among self-identified non-Hispanic White smokers (Table 7), the rs1051730 minor allele frequency of 0.385 in HapMap Utah Residents with Northern and Western European Ancestry sample, and estimated allele effect sizes of Munafo *et al.* (adjusted and unadjusted for CPD).

## Discussion

### TES research opportunities

TES data and banked biospecimens, together with current biotechnologies, offer opportunities for the tobacco research community to identify behavioral, clinical, environmental and molecular factors that may influence cigarette smoke exposures (susceptibility model) and identify molecular factors that may be modulated by cigarette smoke exposures (response model). In particular, the TES can provide existing BOE and BOPH data from a large sample of generally healthy individuals, as well as banked biospecimens for the generation of novel BOE and BOPH. We will conduct biomarker research in the context of an Analysis Consortium that will enhance the TES by adding novel biomarkers and biomarker analyses to elucidate relationships between cigarette smoke exposures and health effects. We will share data with other collaborations engaged in the analyses and meta-analyses of susceptibility and response models. We will deposit data with the repositories designed for genome-wide data per Federal guidance or journal practice and conditional on Human Subjects Committee approval.

Specifically, GWAS using TES PBMC DNA may contribute to the elucidation of relationships between germline variation and self-report and laboratory measures of exposures [38, 39], including genetic loci influencing the non-nicotine tobacco-specific BOE NNAL. Analyses of TES PBMC mitochondrial DNA (mtDNA) via copy number and deletion analysis [40] may enhance knowledge of the factors that influence mtDNA damage [41, 42]. Analysis of PBMC DNA and RNA will provide additional data to examine the effects of cigarette smoking on the PBMC epigenome [43, 44] and transcriptome [45]. Analyses of the plasma and urine proteome [46, 47] and metabolome [48–50], may make a contribution to the developing literature of the impact of tobacco and other exposures defining the *exposome*, an integrated approach to biomarker discovery for exposure and disease paradigms [51]. Validation, integration and extension of these susceptibility and response models can be conducted in independent datasets and in meta-analyses, and may contribute to the development of biomarker panels for diagnostic, prognostic and therapeutic research in tobacco-attributable disease.

There are a number of differences in the landscape of smoking behaviors, tobacco/nicotine products and tobacco control between the time in which the TES was conducted and the present day [52]. These differences include: 1) the prevalence of cigarette use in U.S. adults has declined from ~21 to ~18 %; 2) the regular use of electronic cigarettes has increased in prevalence from 0 to almost 3 %; 3) the annual spending on advertising of tobacco products in the U.S. has declined from an all-time high of $15.4 billion in 2003 to $9.6 billion in 2012; 4) there has been a substantial increase in restrictions on smoking in public places due to increased recognition of harm associated with exposure to second and third-hand smoke; and 5) the passage in 2009 of the Family Smoking Prevention and Tobacco Control Act which prohibited the use of terms in advertising related to “light” cigarettes and created a regulatory framework by which the FDA can evaluate new tobacco products prior to their marketing to the public. Even with these temporal differences, there are several similarities concerning the cigarettes themselves that are of most relevance to the present investigation of cigarette smoking and its impact on BOE and BOPH. These include: 1) despite various changes in cigarette design over the past 12 years, there is no evidence that any of these have resulted in a “safer” cigarette; 2) the amount of tar and nicotine in cigarettes has remained relatively stable since 1993; 3) the most popular brands of cigarettes smoked (see Table 10) remain the same (Marlboro, Camel, and Newport); 4) the effects of exposure to combustible tobacco products both with respect to BOE and BOPH remain the same; and 5) the health consequences of exposure to cigarette smoke (either mainstream or sidestream) including cancer, cardiovascular disease, and respiratory disease remain the same. Since the primary focus of the present investigation is on BOE and BOPH that reside within pathways resulting in negative health outcomes, the TES remains as relevant today as in 2003.

TES biospecimens provide a sample of current smokers powered at GWS to identify the chr15q25.1 nAChR loci associated with BOE (cotinine levels [39, 53, 54], and NNAL [55]). These biospecimens may provide data for future meta-analyses of BOE in both European ancestry and African ancestry samples. TES participants who are current smokers, have smoking topography data, BOE and banked biospecimens are suitable subjects for pharmacogenetic or pharmacometabolic research, e.g., to identify drug metabolizing enzyme and transporter gene associations with existing tobacco-specific BOE, or with as yet undetermined metabolic profiles in 24-h urine. TES biospecimens and data can be used to identify or replicate novel susceptibility or response models, especially in collaborative meta-analyses. Such results may be validated in larger datasets focused on the analysis of tobacco exposures, such as the Population Assessment of Tobacco and Health (PATH) study [56].

### Limitations to the resource

The TES was a multi-site, cross-sectional study with collection sites distributed across the U.S. The sample has limited numbers of individuals with self-identified race other than Black or White, and has limited numbers of individuals with self-identified ethnicity of Hispanic. The diversity in geographical collection is an opportunity to evaluate region as a covariate in both cigarette smoke exposure susceptibility and response-to-tobacco models, e.g., comparing BOE by region or state. However, regional diversity also represents a challenge for future analyses due to potential confounding. Some potential confounders can be measured at a molecular level and used as a covariate in analyses, e.g., principal components of population genetic variation [57] can be evaluated by region or by state.

TES participants with banked biospecimens exhibit small statistically significant differences in demographics and biomarkers compared to TES participants without banked biospecimens. With respect to differences in demographics, participants with banked biospecimens were significantly older and more likely to self-identify as White. The smaller proportion of Black TES participants with banked biospecimens compared to White TES participants with banked biospecimens is consistent with contemporaneous observations in epidemiologic cohorts of reduced willingness to provide consent for future genetic testing in the National Health and Nutrition Examination Survey of 1999–2000 [58], and reduced willingness to provide consent for storage of DNA for future genetic testing in the Baltimore Epidemiological Catchment Area study of 2004–2005 [59], even though the TES was not a representative population-based survey based on national or local sampling. With respect to differences in exposure, smokers with banked biospecimens had increased NE per 24 h and reduced serum cotinine, consistent with the differences observed in demographic characteristics. Despite these small statistically significant differences in demographics and exposure between TES participants with and without biospecimens, TES participants with banked biospecimens can be selected by specific clinical and laboratory criteria to create defined datasets for molecular analyses.

### Use and availability of TES data and biospecimens

The principal intended result of any analysis of the TES is the generation of knowledge related to smoking and health that is shared with the public health community and in the scientific peer-reviewed literature. SRI and the VTHRR agreed on the following principles regarding use of TES data and biospecimens. First, maintain the integrity of the data and samples, i.e., establish infrastructure to track and make the data and biospecimens secure. Second, ensure that potential users of the TES data and/or biospecimens are scientific researchers or organizations focused on the intended analysis goals of the TES, as assessed by education, experience, or by publication track record. Third, include terms in Material Transfer Agreements requiring recipients of data and/or samples to make reasonable efforts to publish the results of studies approved after scientific advisory committee review in the peer-reviewed scientific literature. Under data-sharing guidance for researchers using Federal (e.g., NIH) funds [60–62] and an Office of Science and Technology Policy memorandum [63], scientists who generate molecular data, using array-based or high-throughput genomic technologies are obligated to submit both phenotype and molecular data to qualifying databases.

This is the first time that TES data and biospecimens will be made available to independent scientists in any life sciences area. There is a need for careful, objective scientific analysis of the resource. Consistent with the 2012 recommendation of the U.S. Institute of Medicine to incorporate an independent Tobacco Research Governance Entity [64], SRI has engaged leading experts to form a TES Scientific Advisory Board. This board will provide oversight, review and adjudication of research applications to use the TES data and biorepository resources.

Due to the large size of the TES research resource and the possibilities for integrative analyses, we emphasize our interest in collaborating with individual or groups of investigators, institutions and/or sponsors. Analysis of multiple domains of molecular signatures from TES biospecimens will elucidate the contribution of the genome to exposure susceptibility and the subsequent response of multiple –omic domains to cigarette smoke exposure. Investigators interested in collaborative or independent investigations using the TES data and biospecimens are encouraged to contact the SRI authors.

## Conclusions

The TES research resource represents a sample of 4662 current cigarette smokers and tobacco product and nicotine non-users and includes: behavioral and demographic data; cigarette product characteristics; self-reported clinical data and laboratory-based BOE and BOPH; and banked biospecimens suitable for molecular analyses from >3000 participants. We identified small but statistically significantly greater self-reported measures of cigarette consumption and NE in participants who had consented to contribute biospecimens for banking and future analysis, primarily in self-identified non-Hispanic White smokers, compared to those not contributing biospecimens. The sample of TES participants with biospecimens is statistically powered to provide information on existing susceptibility biomarkers in self-identified Blacks and in self-identified Whites, and represents a well-powered resource to identify novel biomarkers of susceptibility and response to cigarette smoke exposures. The TESAC will seek support to enable research efforts to generate and contribute –omic data to research consortia and to public databases, and findings to the peer-reviewed literature. Such findings will contribute to the understanding of the relationship between cigarette smoke exposures and attributable disease.

## Additional files

Additional file 1:
**Total Exposure Study inclusion and exclusion criteria.** (PDF 75 kb) Additional file 2: Table S1. Biomarkers of exposure in the Total Exposure Study. (PDF 353 kb) Additional file 3: Table S2. Biomarkers of potential harm in the Total Exposure Study. (PDF 191 kb)

## Abbreviations

TES: Total Exposure Study
BOE: Biomarkers of exposure
BOPH: Biomarkers of potential harm
U.S.: United States
VTHRR: Virginia tobacco and health research repository
BMI: Body mass index
FTC tar: Federal trade commission machine-rated tar
CROs: Clinical research organizations
TESAC: TES Analysis Consortium
KEDTA: Potassium ethylenediaminetetraacetic acid
ACDA: Acid citrate dextrose solution A
PBMC: Peripheral blood mononuclear cell
UCSF: University of California San Francisco
HIPAA: Health insurance portability and accountability act
CAL: Clinical analysis laboratory
NE: Total nicotine equivalents
LC-MS/MS: Liquid chromatography-mass spectrometry-mass spectrometry
NNAL: 4-(methylnitrosamino)-1-(3-pyridyl)-1-butanol
NNK: 4-(methylnitrosamino)-1-(3-pyridyl)-1-butanone
COHb: Carboxyhemoglobin
1-OHP: 1-hydroxypyrene
3-HPMA: 3-hydroxy-propylmercapturic acid
4-ABP: 4-aminobiphenyl
Hb: Hemoglobin
GC-MS: Gas chromatography–mass spectrometry
MHBMA: Monohydroxyl-butenylmercapturic acid
DHMBA: Dihydroxy-butyl-mercapturic acid
BRFSS: Behavioral Risk Factor Surveillance System
EIA: Enzyme immunoassay
WBC: White blood cell
hs-CRP: High-sensitivity C-reactive protein
HDL: High-density lipoprotein
LDL: Low-density lipoprotein
BP: Blood pressure
FEV_1_: Forced expiratory volume in 1 second
FVC: Forced expiratory vital capacity
CPD: Cigarette per day
N: Total number
SD: Standard deviation
CARDIA: Coronary artery risk development in young adults
GWAS: Genome-wide association study
NHANES: National health and nutrition examination survey
nAChR: Nicotinic acetylcholine receptor
PATH: Population assessment of tobacco and health
IOM: Institute of medicine
