# Supplementary material for: Total Exposure Study Analysis consortium: a cross-sectional study of tobacco exposures
Source: BMC Public Health. 2015 Sep 7;15:866. doi: 10.1186/s12889-015-2212-5 (PMC4561475; doi:10.1186/s12889-015-2212-5)
Supplement: Additional file 1: — Total Exposure Study inclusion and exclusion criteria. (PDF 75 kb) [file 12889_2015_2212_MOESM1_ESM.pdf]

Additional File 1. Total Exposure Study inclusion and exclusion criteria.

Inclusion criteria were:

1. Males and females, in generally good health, 21 years of age and older (age verified by government issued identification);

2. Able to understand and willing to sign an Informed Consent Form and to comply with study procedures and study schedule;

3. Negative urine pregnancy test for female subjects at Visit 1;

4. Smoking status defined as:

    a. Adult Smokers: regular consumption of a minimum of 1 manufactured cigarette per day during the last 12 months; OR

    b. Adult Non-smokers: no smoking or use of any nicotine-containing product such as snuff, chewing tobacco, patches, lozenges, pills, nicotine-containing water, nicotine gum and/or sprays for 5 years prior to Visit 1 and throughout the study duration.

5. Stable health status (i.e., no major medical events such as diabetic coma, MI, etc) within 14 days of Visit 1, as determined by the site Investigator.

Exclusion criteria were:

1. Age under 21 years;

2. Pregnancy or nursing at Visit 1;

3. Any condition or prior therapy which, in the opinion of the Investigator, would make the subject unsuitable for this study;

Adult Smokers:

    a. Consumption of <1 manufactured cigarette per day during the last 12 months.

    b. Switched brands during the last 3 months prior to Visit 1.

c. Use of a different brand of cigarettes outside their preferred brand tar range at a rate of ≥10% of daily consumption during the last 3 months prior to Visit 1.

d. Use of any nicotine-containing product other than manufactured cigarettes (including roll-your-own cigarettes, bidis, snuff, nicotine inhaler, pipe, cigar, chewing tobacco, nicotine patch, nicotine spray, nicotine lozenge, nicotine pill, nicotine-containing water, or nicotine gum) within 3 months prior to Visit 1.

Adult Non-smokers

a. History of smoking within the past 5 years.

b. History or use of any tobacco- or nicotine-containing product (including cigarette, roll-bacco, nicotine patch, nicotine spray, nicotine lozenge, nicotine pill, nicotine-containing water, or nicotine gum) within 5 years prior to Visit 1.

4. Donation or receipt of whole blood or blood products within 3 months prior to Visit 1.

5. Participation in a clinical study for an investigational drug, device, or biologic within 3 months before, or during, participation in this study unless approved by the study doctor.

6. Any person who is a current or former employee of the tobacco industry, or their first-degree relatives (parent, sibling, child).

7. Any personnel involved with the study at an investigational site.

8. Participation in the same study at a different time or location.
