# Supplementary material for: Total Exposure Study Analysis consortium: a cross-sectional study of tobacco exposures
Source: BMC Public Health. 2015 Sep 7;15:866. doi: 10.1186/s12889-015-2212-5 (PMC4561475; doi:10.1186/s12889-015-2212-5)
Supplement: Additional file 2: Table S1. — Biomarkers of exposure in the Total Exposure Study. (PDF 353 kb) [file 12889_2015_2212_MOESM2_ESM.pdf]

Additional Table 1. Biomarkers of exposure in the Total Exposure Study.

Biomarker[2]	Component(s)	Matrix	Laboratory measurement technology	Related to which Harmful and Potentially Harmful Constituents of Tobacco Smoke [55, 56]
Particulate phase				
Nicotine Equivalents (NE)	Nicotine, cotinine, trans-3'-hydroxycotinine and the corresponding glucuronides	24 hr urine	Liquid chromatography-mass spectrometry/mass spectrometry (LC-MS/MS)	Nicotine or Nicotine (total)[3]
Serum cotinine	cotinine and trans-3'-hydroxycotinine [57]	Serum	Immunoassay	Nicotine or Nicotine (total)[3]
Total NNAL	Total 4-(methylnitrosamino)-1-(3-pyridyl)-1-butanol, and its glucuronide	24 hr urine	LC-MS/MS	metabolite of NNK[4]
NNN[1]	N-nitrosonornicotine	24 hr urine	LC-MS/MS	N-nitrosonornicotine[5]
4-ABP[2]	4-aminobiphenyl hemoglobin (Hb) adducts	Blood	Gas chromatography-mass spectrometry	4-aminobiphenyl (CA)
Gas phase				
1-OHP[2]	Total 1-hydroxypyrene	24 hr urine	LC-MS/MS	polycyclic aromatic hydrocarbons[5]
3-HPMA[2]	3-hydroxy-propylmercapturic acid	24 hr urine	LC-MS/MS	metabolite of acreolein[6]
COHb[2]	carboxyhemoglobin	Blood	Spectrophotometry	Carbon monoxide[7] (RDT)
MHBMA[2]	Monohydroxyl-butenylmercapturic acid	24 hr urine	LC-MS/MS	metabolite of 1,3 butadiene[5]
DHMBA[2]	Dihydroxy-butyl-mercapturic acid	24 hr urine	LC-MS/MS	metabolite of 1,3 butadiene[5]
S-PMA[1,2]	S-phenylmercapturic acid	24 hr urine	LC-MS/MS	metabolite of benzene[8]
Naphthalene[1,2]	aka 2-aminonaphthalene	24 hr urine	LC-MS/MS	2-aminonapthalene[5]
3-OH-B[a]P[1,2]	3-hydroxy-benzo[a]pyrene	24 hr urine	LC-MS/MS	a metabolite of benzo[a]pyrene[5]
o-Toluidine[1,2]	3,3'-dimethylbenzidine	24 hr urine	LC-MS/MS	o-Toluidine[5]

[1]Not described by Roethig *et al* [2], but available in TES data.[2]Biomarker is not tobacco specific. [3]Reproductive or developmental toxicant, RDT, and addictive, AD. [4] Metabolite of NNK, 4-(methylnitrosamino)-1-(3-pyridyl)-1-butanone, a carcinogen, CA. [5]CA. [6]Respiratory toxicant, RT, and cardiovascular toxicant, CT. [7]RDT. [8]CA, CT, and RDT.
