# Supplementary material for: Total Exposure Study Analysis consortium: a cross-sectional study of tobacco exposures
Source: BMC Public Health. 2015 Sep 7;15:866. doi: 10.1186/s12889-015-2212-5 (PMC4561475; doi:10.1186/s12889-015-2212-5)
Supplement: Additional file 3: Table S2. — Biomarkers of potential harm in the Total Exposure Study. (PDF 191 kb) [file 12889_2015_2212_MOESM3_ESM.pdf]

Additional Table 2. Biomarkers of potential harm in the Total Exposure Study [3].

Physiological Function	Biomarker (full name)	Biomarker (acronym), if applicable	Detection Method and Matrix
Cardiovascular	Heart Rate	HR	After being seated for 5’
Cardiovascular	Systolic Blood Pressure	SBP	After being seated for 5’
Cardiovascular	Diastolic Blood Pressue	DBP	After being seated for 5’
Endothelial function	von Willebrand factor	vWF	Enzyme immunoassay (EIA), plasma
Endothelial function	Microalbumin		Immunonephelometry, 24 hr urine
Platelet activation	11-Dehydrothromboxane-B_2	11-DHTB	EIA, 24 hr urine
Hematologic	Hemoglobin	Hb	Bayer Advia 120 automated hematology (AH), blood
Hematologic	Hematocrit	Hct	AH, blood
Hematologic	Platelet count	Plts	AH, blood
Inflammation	White blood cell count	WBC	AH, blood
Inflammation	High-sensitivity C-reactive protein	hs-CRP	Immunonephelometry, serum
Inflammation	Plasma fibrinogen		Photometry, plasma
Metabolism	Uric acid	UA	Roche Hitachi clinical analyzer (RH), 24 hr urine
Renal	Creatinine		RH, 24 hr urine
Renal	Blood urea nitrogen	BUN	RH, 24 hr urine
Lipid metabolism	Total Cholesterol	Total C	RH, serum
Lipid metabolism	High-density lipoprotein cholesterol	HDL-cholesterol	RH, serum
Lipid metabolism	Low-density lipoprotein cholesterol	LDL-cholesterol	RH, serum
Lipid metabolism	Triglycerides		RH, serum
Hepatic	Alanine aminotransferase	ALT	RH, serum
Hepatic	Aspartate aminotransferase	AST	RH, serum
Hepatic	Alkaline phosphatase	ALP	RH, serum
Hepatic	Lactate dehydrogenase	LDH	RH, serum
Hepatic	Total bilirubin	TB	RH, serum
Hepatic	Serum albumin	Alb	RH, serum
Respiratory	Forced expiratory volume in 1 second	FEV1	Spirometry
Respiratory	Forced expiratory vital capacity	FVC	Spirometry
Metabolism	Glucose	Glucose	RH, serum
Oxidative stress	8-epi-prostaglandin $F_{2\alpha}$	8-epi-PG F2α	EIA, 24 hr urine
